# Supplementary figures and images for: sRNA-mediated regulation of gal mRNA in E. coli: Involvement of transcript cleavage by RNase E together with Rho-dependent transcription termination
Source: PLoS Genet. 2021 Oct 28;17(10):e1009878. doi: 10.1371/journal.pgen.1009878 (PMC8577784; doi:10.1371/journal.pgen.1009878)

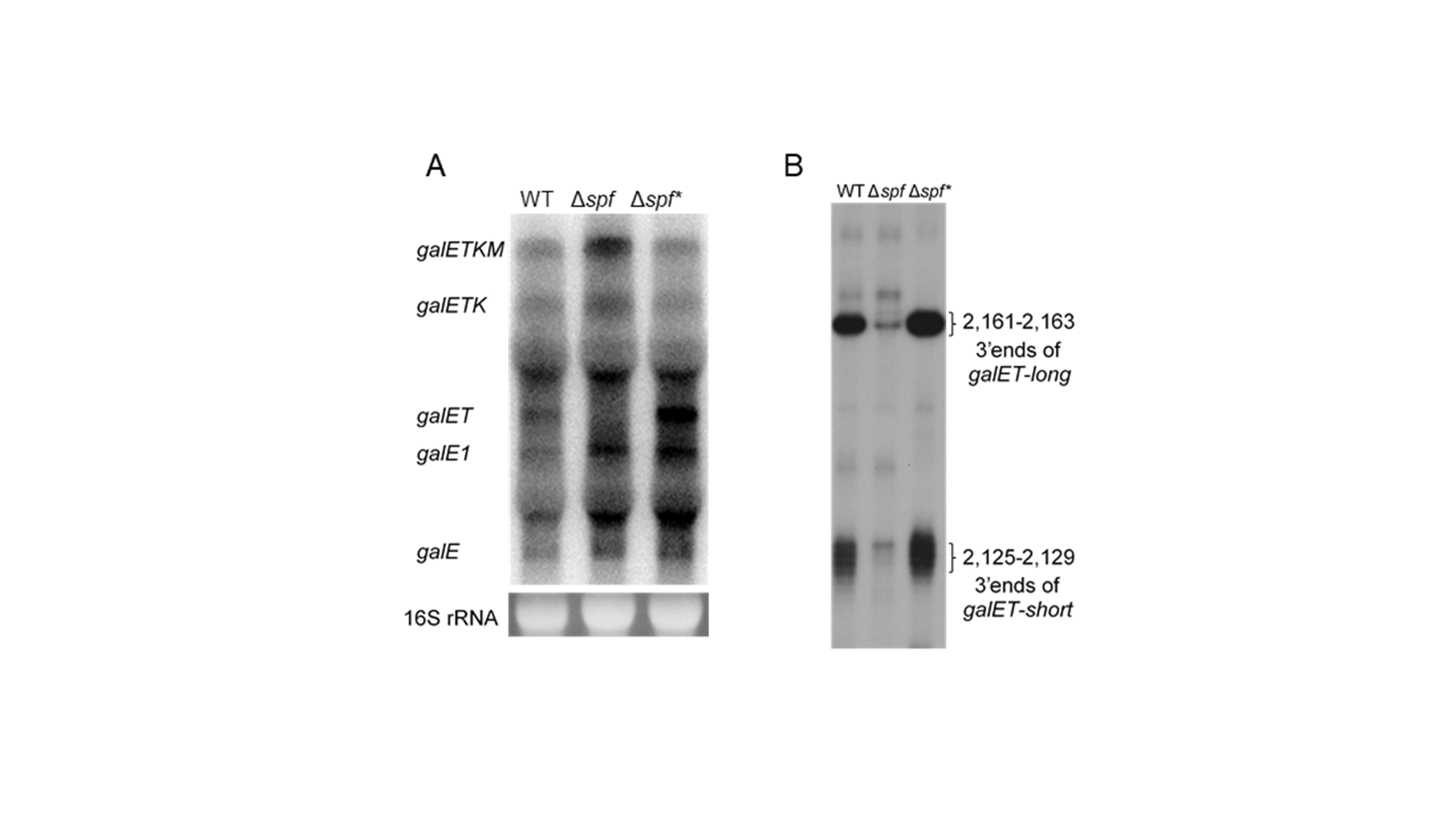

Supplement: S1 Fig — In the absence of Spot 42, particularly the galET band in (A) disappears from the northern blot (lane marked Δspf), as was seen in Fig 1B. Similarly, in Δspf cells, generation of the 3’ ends of galET-short and -long is significantly (~ 95%) inhibited. When Spot 42 was overproduced (spf*), the galET production increased 150% of WT (A), and the production of the 3’ ends of galET-short and -long also increased correspondingly (B). (TIF) [file pgen.1009878.s001.TIF]

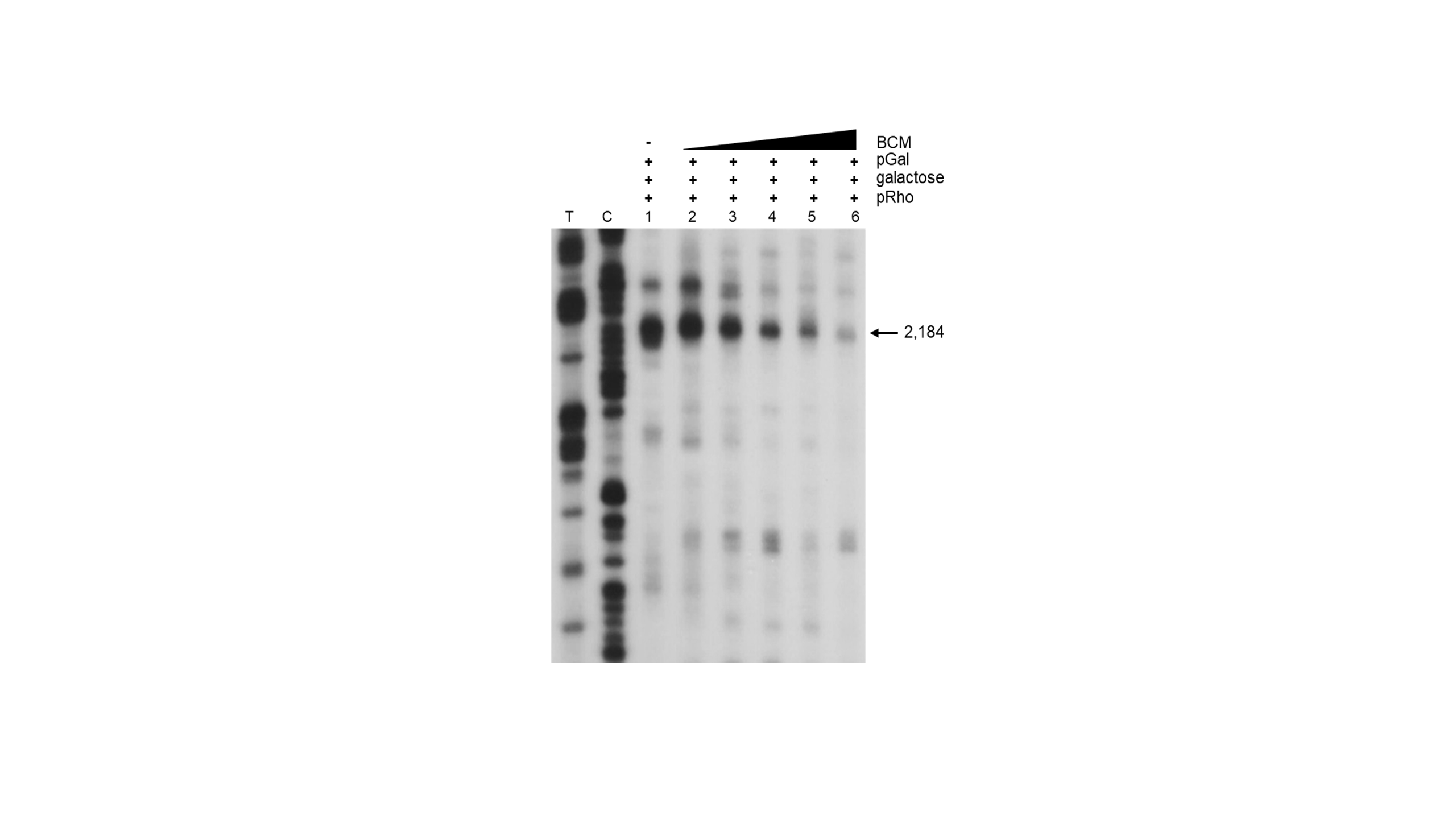

Supplement: S2 Fig — 3’RACE assay of galET mRNA 3’ ends from the cell-free system with pGal, pRho and 0.5% galactose, and either with no BCM (lane 1) or increasing concentrations of BCM (0.1, 0.5, 1, 5 and 10 μg/ml) (lanes 2–6). (TIF) [file pgen.1009878.s002.TIF]

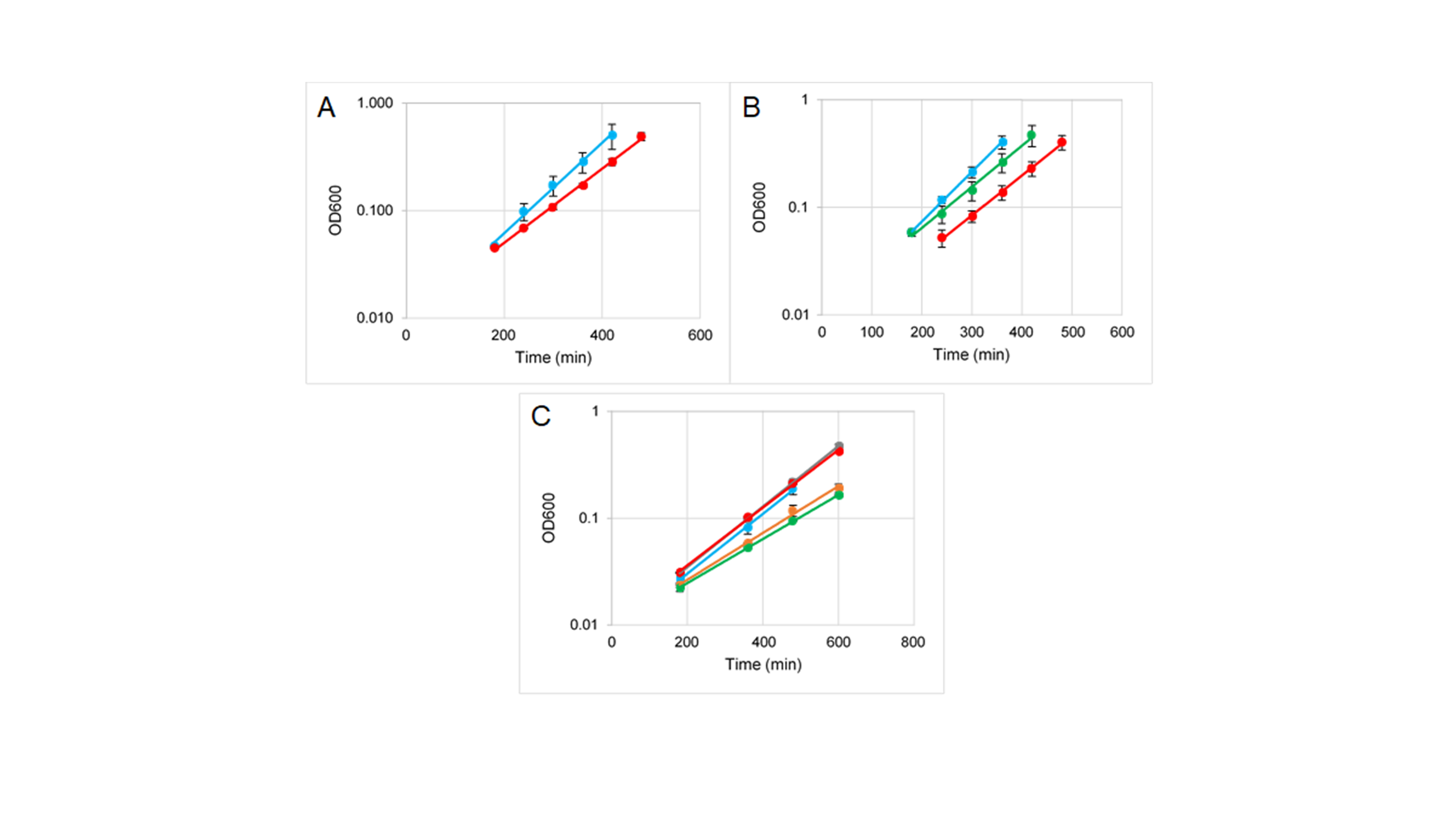

Supplement: S3 Fig — (A) MG1655 (blue) and MG1655 Δspf (red). (B) MG1655Δgal /pGal (blue), MG1655Δgal /pgalMMI-1 (green) and MG1655Δgal /pgalMMII-1(red). (C) MG1655ΔgalΔspf /pGal/pSpot42 (blue), MG1655Δgal Δspf /pgalMMI-1/pSpot42 (brown), MG1655ΔgalΔspf /pgalMMI-1/pSpot42MMI-1 (gray), MG1655ΔgalΔspf/pgalMMII-1/pSpot42 (green) and MG1655ΔgalΔspf /pgalMMII-1/pSpot42MMII-1(red). (TIF) [file pgen.1009878.s003.TIF]

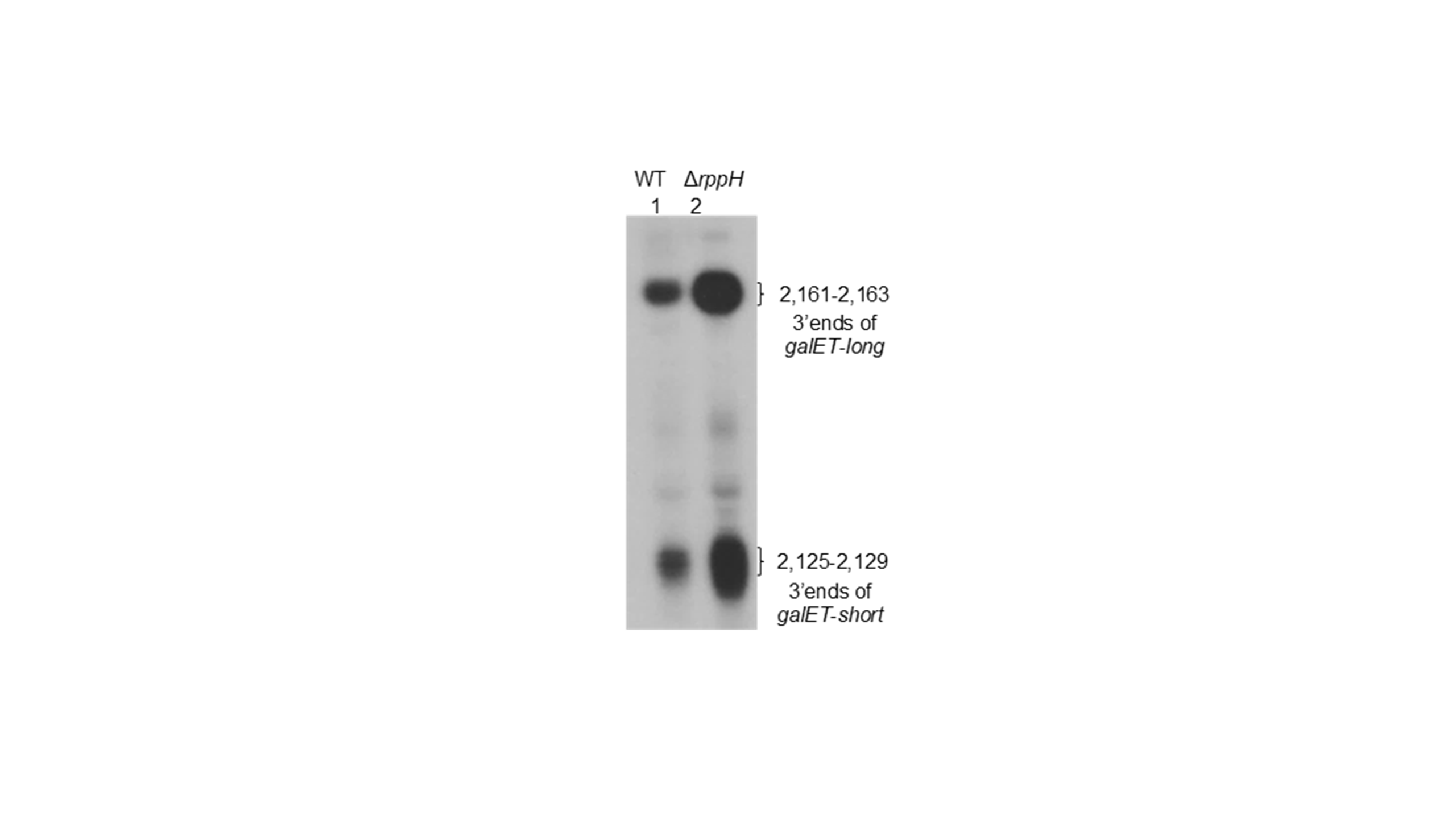

Supplement: S4 Fig — RNase E binds to mono-phosphorylated 5’ end of sRNA. This ability enables RNase E to be recruited to the site of cleavage on target mRNA (1). To see if RNase E cleavage that generates galET-long depends on mono-phosphorylated 5’ end of Spot 42, we assayed the 3’ ends of galET in MG1655ΔrppH strain where the gene for the RNA pyrophosphohydrolase is deleted from the chromosome. Without the RNA pyrophosphohydrolase, most of the 5’ end of RNA remains in di-phosphorylated state [33]. Results showed no difference in generation of the 3’ end of galET-long and -short between WT (lane 1) and MG1655ΔrppH (lane 2) strains. These results demonstrate that the mono-phosphorylated 5’ end of Spot 42 is not a requirement for the generation of galET-long. (TIF) [file pgen.1009878.s004.TIF]
