# Supplementary material for: sRNA-mediated regulation of gal mRNA in E. coli: Involvement of transcript cleavage by RNase E together with Rho-dependent transcription termination
Source: PLoS Genet. 2021 Oct 28;17(10):e1009878. doi: 10.1371/journal.pgen.1009878 (PMC8577784; doi:10.1371/journal.pgen.1009878)
Supplement: S1 Table — (DOCX) [file pgen.1009878.s005.docx]

**S1_Table:**

| **Primer Name** | **Primer sequence 5′ to 3′** | **Use** |
| --- | --- | --- |
| E1 for | ATGAGAGTTCTGGTTACCGGTGGTA | Northern E probe generation |
| E2 rev | TGGGCTTTTTGCAGATCGGTGAGGA |  |
| pSpot42-5'- F | GACGTCGTAGGGTACAGAGGTAAG | Spot 42 cloning in pBRp*lac*-*lacI* |
| pSpot42-3'- R | GAATTCGCGCATCAGGCATTAC |  |
| DMI-1-F | GACGTCTAGGGTACAGAGGTAAG | Spot42 Region I mutagenesis |
| DMI-2–F | GACGTCAGGGTACAGAGGTAAG |  |
| MMI-1-F | GACGTCCGTGGGTACAGAGGTAAG |  |
| MMI-2-F | GACGTCGTACCCTACAGAGGTAAG |  |
| MMII-1-R | CGTGAAGTAAAAGCTCTGAAAGATAGAAC | Spot42 Region II mutagenesis |
| MMII-2-R | CGTGAAGTAAAAGCACTGAAAGATAGAAC |  |
| MMII-3-R | CGTGAAGTAAAAGCAGTGAAAGATAGAAC |  |
| MMIII-1-R | AATATTCAGCCAAATCCGCTTACGTGAAG | Spot42 Region III mutagenesis |
| MMIII-2-R | AATATTCAGCCAAATCGCCTTACGTGAAG |  |
| MMIII-3-R | AATATTCAGCCAAAAGGCCTTACGTGAAG |  |
| MMIII-4-R | AATATTCAGCGAAATCCGATTACGTGAAG |  |
| MMIII-5-R | AATATTCAGCGCTATCCGATTACGTGAAG |  |
| MMIII-6-R | AATATTCAGCGCTTACCGATTACGTGAAG |  |
| 5SF | GAGAGTAGGGAACTGCCA | 5′ RACE for Spot 42 |
| Spf-23n-R-ext | AAAGATAGAACATCTTACCTCTG |  |
| pET-*rnc*-F | TGGTATCATATGAACCCCATCGTAATTAAT | RNaseIII (*rnc*) gene cloning in p*ET-15b* |
| pET-*rnc*-R | TTTTATCTCGAGTCATTCCAGCTCCAGTTT |  |
| Synthetic RNA | UUCACUGUUCUUAGCGGCCGCAUGCUC | RNA oligomer for 3’RACE |
| 3RP | AGCATGCGGCCGCTAAGAAC | RT for 3′ RACE |
| T3 | ACGGTAGCCGTACCGTTGTC | 3′ RACE for *galET* |
| T8 | ATCCATTTTCGCGAATCCGGA |  |
| Gal-HindIII-F | CACCGTTTATGGCGATCAGCCC | *gal* mutagenesis and cloning |
| Gal-MluI-R | GCGTTTTCAGTCAGTATATGACG |  |
| Gal-BamHI-R | CACCCCAGGCTTTACACTTTATGC |  |
| galMMI-1-F | TTGGCTACCCACGCACTCACACCATTC |  |
| galMMI-2-F | CATTTGGCTAGGGTGCCACTCAC |  |
| galMMII-1-F | GTAAGAAATGACTCTGAAAGAAAAAAC |  |
| galMMII-2-F | GTAAGAAATGACACTGAAAGAAAAAAC |  |
| T7-Spot42-for | TTCTGATAATACGACTCACTATAGGGAGAGTAGGGTACAGAGGTAAGATG | Construction of template DNA for *in vitro* transcription of Spot 42 |
| T7-Spot42-rev | TAAAAAACGCCCCAGTCATTACTGAC |  |
| GalE-C-his-T- rev | CATGGTCGTTCCTTAGTGGTGGTGGTGGTGGTGATCGGGATATCCCTG | Construction of pGalE-his, pGalT-his, pGalK-his, and pGalM-his plasmids for western blot analysis |
| GalT-C-his-T- rev | GATATCGCTGACTGCGTGGTGGTGGTGGTGGTGGCGCAAACGCTCTGC |  |
| GalK-C-hisT- rev | CGGGAGTTTCGTTCAGTGGTGGTGGTGGTGGTGGCACTGTCCTGCTCC |  |
| GalM-C-hisT- rev | GAGGGCGTAACATTAGTGGTGGTGGTGGTGGTGCTCAGCAATAAACTG |  |
