# Supplementary material for: sRNA-mediated regulation of gal mRNA in E. coli: Involvement of transcript cleavage by RNase E together with Rho-dependent transcription termination
Source: PLoS Genet. 2021 Oct 28;17(10):e1009878. doi: 10.1371/journal.pgen.1009878 (PMC8577784; doi:10.1371/journal.pgen.1009878)
Supplement: S2 Table — (DOCX) [file pgen.1009878.s006.docx]

**S2 Table**

| **Strain** | **Genotype** | **Reference** |
| --- | --- | --- |
| MG1655 | *F^-^, lambda^-^, rph-1* | ATCC 47076 |
| MG1655Δ*spf* | Δ*spf (*Spot 42 knock out) | [1] |
| MG1655Δ*gal* | Δ*gal (gal operon gene* knock out) | [2] |
| MG1655Δ*gal*Δ*spf* | Δ*gal* Δ*spf (gal operon and* Spot 42 gene knock out) | In this study |
| MG1655Δ*gal*Δ*rppH* | Δ*rppH (rppH* gene knock out) | In this study |
| MG1655Δ*gal*Δ*rnb* | Δ*rnb (*RNase II gene knock out) | In this study |
| MG1655Δ*gal*Δ*rnr* | Δ*rnr (*RNase R gene knock out) | In this study |
| W3110 | *F^-^ lambda^-^ IN (rrnD-rrnE)1 rph-1* | CGSC#: 4474 |
| W3110 Δ*pnp* | Δ*pnp (*PNPase gene knock out) | In this study |
| GW10 | W3110 *zce-726*::Tn*10* | [2] |
| GW11 | W3110 *zce-726*::Tn*10 rng*::*cat* | [2] |
| GW20 | W3110 *zce-726*::Tn*10 rne-1* | [2] |
| NHY312 | (*proBlac*) *ara gyrA thi zic-501*::Tn*10 rnpA*_ | [2] |
| NHY322 | (*proBlac*) *ara gyrA thi zic-501*::Tn*10 rnpA49* | [2] |

**References**

1. Wang X, Ji SC, Jeon HJ, Lee Y, & Lim HM (2015) Two-level inhibition of *galK* expression by Spot 42: Degradation of mRNA mK2 and enhanced transcription termination before the *galK* gene. Proceedings of the National Academy of Sciences of the United States of America. 112(24):7581-7586.

2. Wang X, et al. (2014) Expression of each cistron in the gal operon can be regulated by transcription termination and generation of a *galK*-specific mRNA, mK2. Journal of bacteriology 196(14):2598-2606.
