# Supplementary material for: sRNA-mediated regulation of gal mRNA in E. coli: Involvement of transcript cleavage by RNase E together with Rho-dependent transcription termination
Source: PLoS Genet. 2021 Oct 28;17(10):e1009878. doi: 10.1371/journal.pgen.1009878 (PMC8577784; doi:10.1371/journal.pgen.1009878)
Supplement: Attachment — Submitted filename: Response to reviewers.docx [file pgen.1009878.s007.docx]

Reviewer's Responses to Questions

**Comments to the Authors:
Please note here if the review is uploaded as an attachment.**

Reviewer #1: This is a beautifully written manuscript dealing with the regulation of the E. coli *gal* operon by Spot 42, an sRNA. The findings are surprising and quite novel. Spot 42 promotes Rho-dependent termination at galK as well as degradation of the galK transcript. Amazingly, these effects are performed by different SpoT 42 sequences, both complementary to galK mRNA.

Critique:

1. There are several typos that need correcting.

We have edited the manuscript more carefully now.

2. Eliminating one of the two SpoT activities does not increase the activity of the other, suggesting that the substrate mRNA is in excess. It may suggest that SpoT has two structures that are not in equilibrium. Is anything known about the structure of this sRNA?

We are happy that the Reviewer accepted our inference that the substrate availability may not be a limiting factor (l.364-71). That Spot 42 might have two structures not in equilibrium is an excellent alternate explanation, which we mention briefly (l.364-71). We did not elaborate on this point since not much is known about the structure of this sRNA.

3. The overall efficiency of SpoT processing is very low. I wonder how a SpoT could affect cell growth. And whether it plays a significant role in Gal regulation.

To the extent tested, Spot 42 appears to offer significant growth advantage. The effect is not huge but even a small help can be of evolutionary advantage. The conserved nature of Spot 42 gene attests to this notion. Many sRNAs usually are without a phenotype unless overproduced or cells are appropriately stressed.

4. If would be useful to indicate the extent of processing under the gel lanes (Fig 1).

We mentioned the mRNA band intensities in the text (l. 123-25).

5. Fig 3C needs significance calculations - what is Fig3D looking at? Also, what does the time course mean – it the values are quite variable.

We have removed Fig 3B and C from the manuscript and have added a new data in Fig 5, which we believe is more direct and clearer. The new data suggest that the *galET-short* is generated by RDT in the first place and that the 3’-end of the terminated product is processed to the 3’ ends of *galET-short* by RNase III (New Fig 5 B*)* (l. 274-79). In the previous version of the manuscript in old Fig 4C, the bands labeled 2,121 upon reexamination turned out to be the bunch 2,125-2,129, identical in size to the *galET-short* transcripts*.* We apologize for the oversight.

6. Fig 4 Is the lettering correct? The band at 2184 doesn’t correspond to anything in vivo. What does this figure add to understanding the mechanism of action of SpoT? Unless you can explain why it isn’t seen in vivo.

The most intense band seen in *in vitro* transcription-translation system is at 2184. That the terminated product is not spurious is indicated by the position of the termination event after the *rut* site, and dependence of the termination on Spot 42 and Rho. That the 2184 species is not recorded in vivo can be understood from our new results that RNase III cleaves the transcript of 2184 to generate the *galET-Short* (New Fig. 5B). The processing by RNase III apparently happens readily.

7. Fig 5 – statistical significance not given.

We have also removed Fig 5 from the revised manuscript and added a new Fig 5. We believe the new Fig 5 presents a clearer demonstration of Spot 42-controlled RDT, and further processing of the terminated product by RNase III that generates *galET-short* (New Fig 5B).

Reviewer #2: I have reviewed the manuscript by Jeon et al. submitted to Plos Genetics. The manuscript basically reports investigation of mRNA metabolism originating at the gal operon in E. coli. Specifically, the authors deal with post-initiation processing of RNA and transcription termination, and the regulation of these events. More than 50 years ago, a phenomenon was discovered, called Natural Polarity, which is decreased expression of distal cistrons compared to the expression of promoter proximal cistrons in a polycistronic operon. Regulations both at the transcriptional and translational level have been independently suggested to be responsible for natural polarity but the exact mechanism(s) of natural polarity remained unproven for decades. For the first time, by in depth analysis of the gal operon in E. coli the current authors have demonstrated that the phenomenon is complex and occurs at different levels: transcription termination, RNA processing and translation. A forerunner paper analyzing natural polarity was published in 1980s in the his operon of Salmonella although determining the precise mechanisms was not possible at the time because of lack of modern technologies. In the current manuscript, Jeon et al. showed how a small RNA participates in creating natural polarity in all three steps – transcription termination, RNA processing and translation. It is also noteworthy that involvement of small RNA in Rho-mediated transcription termination is novel.
In gal, the natural polarity is condition dependent – only shown in the absence of cAMP and has huge physiological implications.
The experiments are done in meticulous details with clear and convincing results. Importantly, the authors also nicely correlate the implications of the mechanisms with physiological experiments. I strongly recommend that the manuscript should be published as soon as possible. However, I suggest that several minor editorial corrections before publication. They are:

Line 63. Mention the 3’ endpoint if known.

As suggested, the 3’endpoint is added (l.63).

Line 101. Delete the phrase “rather than by new synthesis”

As suggested, the phrase has been deleted (l.102).

Line 170. It appears that region III of the small RNA does not have a role. The authors may speculate why not.

We provided a basis for how region III could be occluded from pairing with mRNA in our model (Fig 7) (l.802-05).

Lines 241-3. Bicyclomycin does not inhibit Rho activity completely. It is suffice to see a partial effect of the drug is expected. It is not necessary to invoke another factor.

We deleted the speculation as suggested.

Line 248. Use the phrase ‘in the presence of’ of ‘adding’.

Done (l.239-40).

Line 251. Same. ‘In the presence’ of plasmid pRho instead of ‘adding’.

As suggested, the phrase has been changed (l.242).

Lines 260 and 267. Same. ‘In the presence’ of pGal, etc.

As suggested, the phrase has been changed (l.245).

Line 270. ‘in the galK gene sequence’.

The part of the text has been removed in the revised manuscript.

Line 300. …. at ‘the’ galT…

Inserted (l.251).

Line 313. Here ‘we’ also found….

Inserted (l.284).

Line 317. “Increased”?

Increased changed to “would increase’ (l.288).

Line 327. It is better to say ‘…. Expression facilitating the anabolic role of the pathway’.

As suggested, the phrase has been changed (l.298).

Starting line 328. Great experiments showing the physiological relevance of spot 42 in gal operon expression.

We are happy that the Reviewer liked the experiments.

Line 352. Extend the sentence ‘E. coli .. at post transcription initiation level.

As suggested, the sentence has been extended (l.324)

Line 373. The unknown enzyme could be RNase II as shown by the authors (ref. 4).

In the revised manuscript, RNase III appears to the enzyme that is responsible for the generation of the 3’ ends of *galET-short* (new Fig 5B). Fig 5 shows that RNase III processed the 3’ end from 2,184 (Fig 4A and B), which is the product of RDT, to the 3’ ends of *galET-short* at 2,125-2,129 (previously misinterpreted as 2,121, we apologize) (l.274-79).

Lines 374-377. See comments on lines 241-243.

We deleted the speculation, have removed Fig 3B and C from the manuscript, and have added a new data in Fig 5, which we believe is more direct and clearer. In the revised manuscript, RNase III appears to the enzyme that is responsible for the generation of the 3’ ends of *galET-short* (new Fig 5B). Fig 5 shows that RNase III processed the 3’ end from 2,184 (Fig 4A and B), which is the product of RDT, to the 3’ ends of *galET-short* at 2,125-2,129 (previously misinterpreted as 2,121, we apologize) (l.274-79).

Line 410. I agree. PNPase acts the same way as RNAse II does.

Both the enzymes are major exoribonucleases and are essential components of the RNase E-degradasome complex.

Line 433. …in ‘polycistronic’ gene regulation (l.463).

‘polycistronic’ inserted (l. 402).

Discussion. It is bit long, and may be somewhat condensed by removing a few of the ‘remains to be investigated’ topics.

We have trimmed the Discussion a bit. Only four topics are discussed, the last one was encouraged by this referee, as stated in the comment below.

Discussion. I suggest that the authors mention the relationship of cAMP and polarity in gal that I discuss above simply because the relationship has been unraveled in the paper.

We thank the reviewer for this valuable suggestion. We have included a paragraph explaining the relationship of cAMP and polarity in *gal* operon in the revised manuscript (l. 403-408).

Reviewer #3: The manuscript by Jeon et al investigates regulation of the E. coli gal operon by Rho, RNaseE, and a small RNA Spot 42. This work builds on previous observations published in 2014 and 2015. The authors show that binding of Spot 42 to the gal mRNA leads to Rho-dependent termination (that was already shown by the authors) and RNase E cleavage at two closely-spaced sites.
In my opinion, the insights provided by this work fall short of the PLOS Genetics standards.
Binding of Spot 42 to the TIR of galK is expected to inhibit translation, making RNA susceptible to Rho and RNA degradation. In their previous PNAS paper, the authors proposed just that. The new data presented in this work are (i) effects of mutations in mRNA, sRNA, and compensatory mutations on the formation of two mRNA species and (ii) the involvement of RNaseE, but not RNaseP or G, in mRNA processing. With respect to Rho, the authors add a lot of small new pieces, including in vitro coupled assay in which Rho is expressed. However, nothing principally new is shown and their previous conclusions do not change. It is well established how Rho works.

The new finding here is that *galET* comprises two species. Although Rho involvement in the generation of *galET* was known, the new result begged the question whether Rho is involved in generating one or both the species. This study is also the first to reproducing Rho-dependent transcription‑termination promoted by Spot 42 *in vitro*. A noteworthy bonus from this *in vitro* assay is the finding that termination occurred immediately after a putative RNA polymerase pause site. Finally, we report a brand new finding that a double-strand specific endoribonuclease, RNase III, is involved in the processing of sRNA-regulated products.

On that note, the authors should brush up on Rho literature – papers they cite on the mechanism of action of BCM and Rho itself do not reflect current knowledge in the field, and their discussion does not incorporate recent findings. Similarly, the collection of transcription-translation references is really odd.

We tried to update references on Rho and revised the collection of references on transcription and translation.

By contrast, data on RNase E are limited to one gel with a ts mutant. If the authors want to make their study impactful, they should investigate this aspect of the regulatory mechanism.

We did not elaborate on RNase E results because they were clear-cut. Instead, in the revised ms. we concentrated on the role of Rho, where the results were not as clear.

With respect to Spot 42, the authors should inhibit translation of galK by other means (many possibilities) to distinguish different aspects of its contribution to gal control. The authors conclude that Spot 42 “directly” controls termination based on data in Fig. 4 – this is impossible to tell in the assay used, as all RNA synthesis goes up upon the addition of Spot 42 RNA, not only the species authors mention. In this and other assays, a normalization control is essential.

It is true that all bands increase with increasing Spot 42 concentration in Fig 4 but the magnitude of increase is not same in all. It is by far the most for the bands at 2184 and at 2,125-9 (Fig 4B, lanes 3-5)

Some aspects of other assays are puzzling. For example, in Fig. 3, the authors argue that a small effect of Rho inhibitor BCM after extended incubation is supportive of their conclusions. Rho terminates 20% genes in E. coli, and BCM leads to a lot of secondary effects; particularly after 1 hour. In this case, error bars would be critical but are absent.

We have removed Fig. 3B and 3C from the manuscript and have added new data, which are direct and clearer (Fig 5). The figure in the revised version of the manuscript suggests that the *galET-short* is generated by RDT and that the 3’-end of the RDT generated transcript is processed to the 3’ ends of *galET-short* by RNase III (New Fig 5B) (l.274-79).

The authors mention the use of strain with a non-functional Rho – they must elaborate, as rho is an essential gene in E. coli unless RNase H/UvsW is overexpressed. Comparing reporter fusions to tRNA-Arg gene +/-rut makes no sense. One should do RT PCR probing regions before and after terminator. And the effects presumably due to termination are tiny! There are tens of well-characterized mutants in Rho and NusG that could be used to probe the role of Rho. Making physiological arguments based on plasmids that express regulatory factors is not acceptable. MG1655 can be genetically modified as needed.

We have also removed Fig. 5 of the original manuscript and added a new Fig. 5. We believe the new Fig. 5 will present a clearer picture of Spot 42 control of RDT, as stated in the answer to the previous comment. Because we used a cell-free system, physiology is less of a concern.

Most importantly, the authors discuss possible effects of uncoupling of transcription and translation and the roles of small RNAs. There is no doubt that sRNAs have a lot of targets and control expression of many genes. But the effects reported here are small, not necessarily direct, and need to be investigated in more detail.

We have dealt with two pleiotropic proteins (RNase E and Rho) here and were able to come up with some clear findings. RNase E results are quite dramatic (Fig 3, lanes 8,10) and we believe the Spot 42 mediated RDT results are also equally dramatic (Fig 4B, lanes 3-5). We sincerely hope that the new data will be more acceptable to the reviewer. We do not argue that the investigations could have been done in more detail.

Reviewer #4: In this work, Jeon and Lee et al. build upon previous analyses of regulation in the well-studied E. coli gal operon. The authors present the interesting finding that both RNase E and Rho, in conjugation with the sRNA, Spot 42, control formation of alternative forms of the galET sub-transcript (long and short forms, respectively). In particular, base-pairing between Spot 42 region I and the gal transcript favors generation of galET-long, and base-pairing between Spot 42 region II and the gal transcript favors generation of the galET-short transcript. galET-short generation is dependent upon Rho factor activity, whereas galET-long generation is dependent on RNase E activity. Finally, through specific base-pairing interactions, Spot 42 is predicted to help direct metabolites to anabolic pathways during growth in minimal medium. Overall, this work should be of interest to the field, but the manuscript requires both major (including one experiment) and minor revisions as outlined below.

The major revisions needed for this work center around three points. The first point is that information about biological replicates and numbers of blots represented by those presented should be included in the manuscript. The second point centers around Spot 42-mediated, Rho-dependent termination in vivo and whether Fig 5 is sufficient to establish this mechanism, including a need for information about statistical tests used. It is unclear if Spot 42 region I also contributes to Rho-dependent termination from the data presented (i.e., whether RDT may still be promoted via Spot 42 region I, but processing of the gal transcript to galET-short fails without region II base-pairing). Finally, the data presented in Fig 6 seem to test a prediction that was already known to be true rather than directly testing the contributions made by regions I and II to Gal protein level tuning to add biological relevance to the differential processing mechanism described in this work.

Major revisions:

1. The authors do not provide clear statements about the number of biological replicates used to obtain data presented in the figures (e.g., describing the number of replicate blots representative of blots shown represent). This information is especially important for instances where differences are modest and, as such, should be included. In the same vein, it is also not clear where the data for Fig 3D were derived. In particular, were the mean and SD determined from the intensities in Fig 3C? It may be more informative to show comparisons at each time point (e.g., as a line graph), using p-value adjustments for multiple comparisons (error bars should also be included on each point in Fig 3C—the comparisons would, thus, be between galET-short values at each time point or galET-long values at each time point only, rather than between the two isoforms at each time point). As shown in the same figure, the decrease of galET-short over time seems unclear (in fact, from 10 to 20 minutes the level seems to be increasing; the level at 30 minutes is similarly higher than at 10 minutes, and the levels at 40 and 50 minutes seem to be similar to that at 10 minutes). The authors should also mention that the concentration of BCM used is sub-inhibitory and thus is unlikely to completely abrogate RDT. Finally, the authors should provide an explanation for this ambiguous galET-short depletion over time in the text.

In the revised ms. Fig. 3 has been revised in the vein suggested by the Reviewer. We removed Fig. 3B and C, where BCM showed partial effect. Reviewer#2 pointed out also that BCM inhibits Rho partially. Our experience to push BCM was exactly as stated by Reviewer#3- increasing BCM concentration or reaction time starts showing non-specific effects. For these reasons, we took recourse to a cell-free transcription-translation system where the data are more direct and clearer (Fig 5). The figure in the revised version of the manuscript suggests that the *galET-short* is generated by RDT requiring Spot 42 and that the 3’-end of the RDT generated transcript is processed to the 3’ ends of *galET-short* by RNase III (New Fig 5B) (l. 274-79).

2. The authors should revise figure captions to clearly define the data shown in the figures (i.e., with the exception of Fig 3, it is unclear that bars or points represent a mean with error bars representing SD [how many replicates] or some other metric of variability). In Fig 5, are error bars shown for pHL2000? In the same figure, statistical tests should be performed and reported for the comparison of pHL2000 and pHL2184 (all panels). Are these differences significant? If these differences are not (B and E) or are (C and D), it is unclear what this figure adds to the manuscript. Depending on results of the statistical analysis (or if a statistical analysis cannot be performed), the authors should consider removing the figure (and editing in the text for this modification accordingly).

We agree that the results of Fig 5 of the original manuscript were unconvincing, especially without rigorous statistical analysis. As suggested, we removed those results and provide new Fig 5 that shows clearly the Spot 42 control of RDT and generation of *galET-short* at 2,125-2,129 (previously miscalculated as 2,121, we apologize) by RNase III processing of the 3’ end at 2,184 (Fig 5B, lanes 3, 4) (l. 274-79).

3. Nothing presented in Fig 2, 3, 4, and 5 suggests that some level of RDT cannot be accomplished through Spot 42 region I hybridization (or that region II hybridization alone is sufficient for RDT), just that the short isoform does not accumulate in region II variants. Unless an experiment that determines whether region I/II variants disrupt RDT can be performed, region I hybridization should be depicted in Figure 7A; the possibility of region I contributions to RDT should also be acknowledged in the text.

We added region I hybridization in Fig 7A and state that “We note that from our present results the possibility of region I hybridization contributing to some level of RDT cannot be ruled out.” (L.344-46).

4. The authors seem to make an assumption (given the data presented in Fig 6) that defective Spot 42 base-pairing (Table 1) leads to an altered (reduced) metabolite flux toward an anabolic pathway (possibly reduced metabolite moving toward UDP-glucose/galactose) through Gal protein fine-tuning. However, such specific data is lacking in the manuscript and would make a substantial contribution to Fig 6 (i.e., western blots of Gal protein levels with ectopic expression of Spot 42 [and region I and II variants, along with associated suppressors]).

We appreciate the comment but we could not address because it would have involved significant time and resources. Instead, we considered clarifying the basis of the generation of *galET-short* to be more germane to our paper and concentrated our efforts in that front.

Minor revisions:

Ln 31: Use “Rho” in place of “the Rho factor”.

As suggested, Rho factor was replaced with Rho throughout the revised ms.

Ln 83: Put commas between Spot 42: “The sRNA, Spot 42,…”

Inserted (l.83).

Ln 98: Delete the comma after reference 24.

Deleted (l.98).

Lns 100-2: It is unclear what the authors mean by new synthesis not being required for the two different 3’ ends (new synthesis would be required [galET-long cannot be generated from galET-short, and it was not concluded that galET-short can be generated from galET-long]).

We apologize for the confusion, the phrase ‘rather than by a new synthesis’ has been deleted in the revised manuscript (l.102).

Lns 107-10: Please add a reference for Spot 42 remaining maximal in LB (especially w/o glucose) at 0.6.

As suggested, the reference has been added (l.110).

Lns 134-6: This sentence (“Using 3’ RACE…”) seems like it would be better with the following paragraph (beginning at Ln 137).

Changed. “Using 3’RACE…,” now starts the sentence in l.142.

Ln 212: This section caption is especially uninformative. Consider changing to: “Both RNase E and Rho serve a function in the mechanisms generating unique galET 3’ ends”

The section caption has been changed to “RNase E is involved in the generation of *galET-long* 3’ ends” (l.215). We hope this shorter statement is less passive and informative enough and would be acceptable.

Ln 231: Consider using: “To test whether…” vs. “To test if…” here and elsewhere.

Changed (l.223).

Ln 235: Perhaps it would be better to say “We found that…” vs. “The results showed that…” (Less passive).

Changed (l.123, 217).

Ln 237: This can be part of the previous paragraph; the line space is not necessary.

The line space has been removed.

Ln 238: This may be changed to: "In particular, as a functional Rho is present in the rnets mutant grown at a non-permissive temperature, the drastic reduction in galET-long levels indicates Rho does not serve a significant function during the generation of this transcript isoform."

This part of the manuscript has been deleted in the revised version.

Ln 241: Consider changing "...but this requires RNase E" to "... in an RNase E-dependent manner"

This part of the manuscript has been deleted in the revised version

Ln 246: Consider using “The cell-free system contains all…” instead of “The cell-free system is supposed to contain all…”

The sentence has been changed as suggested (l.236).

Ln 273: It seems appropriate to speculate that RNase E may serve some function generating the 2,121 and 2,166 nucleotide RNAs in the text; the BL21(DE3) cells still produce a functional RNase E (vs. the less functional truncated RNase E variant from BL21 Star [DE3]).

This is a good suggestion. However, we could not find any consensus sequence of the RNase E cleavage sites (RNWUU (R = G or A, W = A or U, and N = any nucleotide.), Chaa et al., 2017). In addition, the new revised Fig 5 in the manuscript suggests RNase III is the enzyme that is responsible for the generation of the 3’ ends of *galET-short* only. The new Fig 5 shows that RNase III processed the transcript with the 3’ end at 2,184 (Fig 4A and B), which is the product of RDT, to the 3’ ends of *galET-short* at 2,125-2,129 (previously misinterpreted as 2,121, we apologize) by endo-nucleolytic cleavage (New Fig 5B) (l. 274-79).

Ln 276: Double-check reference formatting (other references are in brackets)

We have edited the manuscript more carefully now.

Ln 298: The authors should explain the properties of the rho::AmpR allele. Is the rho nonfunctional? Rho is essential; therefore, this mutation is unlikely to produce a nonfunctional variant of Rho. In what way do the properties of rho::AmpR differ from rho15 (Ts)?

We have removed Fig 5 and associated text where the above-mentioned strain was used.

Ln 308: This would be a good spot to introduce the supplemental data on exoribonucleases (vs. introducing this particular new data in the discussion). Although, the results are negative, as noted in the manuscript discussion (should also be acknowledged at this line in the text if the figure is added here), these RNases have redundant function (double mutants are not viable). It may be worthwhile to speculate here on the function of RNases toward dsRNA (such as RNase III) for generation of galET-short. The point could be made that some RNase is necessary to generate galET-short (the transcript from RDT alone would produce a transcript that is too long to be this isoform). This point would be parallel to the section beginning at Ln 399 in the discussion.

We are grateful to this referee for this ingenious suggestion, as it was right on the money. We did test the possibility of RNase III action, which clarified the picture on the generation of the 3’ end of *galET-*short tremendously. The supplemental data on exoribonucleases has been replaced as the new Fig 5A in the revised manuscript (l. 268-73).

Ln 313: I believe the line should read: "Here we also found...."

The line has been corrected (l.284).

Ln 330: change to “…where both anabolism and catabolism of galactose are required.”

The line has been corrected (l.300)

Lns 339-41: Consider editing to: “In exponential growth phase, the doubling times of cells encoding galMMI-1 or galMMII-1 (SI Appendix, S1 Fig B) were 22 and 26 % longer than those encoding the WT gal sequence, respectively (Table 1).”

The line has been edited and updated (l.310-12).

Ln 386: Although the authors present data suggesting the 5’ phosphorylated state of Spot 42 state does not significantly contribute to RNase E-mediated gal transcript processing, it does not rule out the potential internal entry (5’ bypass) pathway also noted in reference 43 (Bandyra et al. 2012. Mol Cell 47:943-53). As the 5’-dependent pathways seem to have unclear importance (or possibly be redundant to the internal entry pathway) for Spot 42-promoted RNA processing, it seems relevant to note the possibility that this alternative pathway makes a contribution to gal transcript processing in the text.

We have now added the possibility of RNase E using the direct entry pathway (l.358).

Ln 775: Delete the comma at the end of the line.

Deleted.

Lns 799-801: The mutation names should match what is shown in the figure (i.e., these can be: “GW20 (amsts [rnets]…”

This part of the manuscript has been deleted in the revised version.

806: Colors of the bars should be noted with respective transcript isoforms.

This part of the manuscript has been deleted in the revised version.

Ln 809: Change the figure panel lettering (and corresponding caption and spots in the text) such that the blots are A-C (look at Ln 813; the ladders are in all three blots, but not B).

The figure panel lettering (l. 756, 760 and 763) has been corrected now. The ladders for the blots are mentioned correctly in the revised manuscript. (l.759, 763).

Ln 817: I believe this is a sliding window GC-content plot; it is customary to say this is what is being depicted and the window size being used to determine GC-content (in the figure caption and text).

We apologize for not mentioning the size of the sliding window in the GC-content plot. A sliding window of 70 was used to plot the GC content; it is now added to Fig 4C caption (l.769-70).

Ln 823: The word “the” should be lowercased. Also, please decide on a consistent choice for capitalization state of “lane” in the caption.

As suggested, the changes have been made (l.773-75).

Ln 830: “were taken…” instead of “was taken…”

Corrected.

Ln 834: Technically, the figure does not assay gal expression. The caption should reflect tuning of protein levels toward an anabolic pathway.

As suggested, the caption of Fig 6 has been modified (l.779).

Ln 865: The color gray should be lowercased. Also, depending on formatting, these text colors may not be included in the final manuscript version of the manuscript.

Corrected. We will address at the proof stage the formatting issue with colors.

Ln 886: Figure S4 is not referenced anywhere in the text. Please make reference to this figure where appropriate.

We apologize, we have added S4 Fig reference in the revised manuscript (l.357).

Ln 935: The font is difficult to read on Fig 6B. It would be more informative to show the individual growth curves (vs. mean and SDs). We have increased the size of Fig 6B. Individual graphs and the letters can now be seen easily.

Do the authors have data points for LB+0.5% galactose (Δspf) beyond 500 minutes? Sorry, we do not.

Ln 958: It would be useful for the authors to show a representation of the assumed cleavage events leading to galET-short in the model (perhaps just by noting “RNase?” in figure panel 7B). It would also be helpful if “RNase E/RNase?” (instead of RNase E alone) cleavages were shown closer at the relevant ends of galET-long and -short (it is unknown precisely where the cleavage/processing events happen, so drawing the cleavage at relevant sites would not be any less inaccurate).

Since we found that RNase III is involved in the generation of products of *galET‑short*, we have now included RNase III/RNase in the model Figure. We mention in the legend that RNase III alone may not be doing the processing, other RNases might also be involved (Fig. 7C) (l.346-48).
