# Supplementary material for: sRNA-mediated regulation of gal mRNA in E. coli: Involvement of transcript cleavage by RNase E together with Rho-dependent transcription termination
Source: PLoS Genet. 2021 Oct 28;17(10):e1009878. doi: 10.1371/journal.pgen.1009878 (PMC8577784; doi:10.1371/journal.pgen.1009878)
Supplement: Attachment — Submitted filename: Response to Reviewers 2.docx [file pgen.1009878.s008.docx]

Reviewer's Responses to Questions

**Comments to the Authors:
Please note here if the review is uploaded as an attachment.**

Reviewer #1: The revised manuscript is much improved and answers the criticisms to this reviewer's satisfaction.

We are happy that the Reviewer approved the revisions and recommended the manuscript for publication.

Reviewer #2: The authors have satisfactorily revised the manuscript adequately responded to the criticisms of the reviews. I recommend its publication in Plos Genetics.

We are happy that the Reviewer approved the revisions and recommended the manuscript for publication.

Reviewer #3: A revised manuscript by Jeon et al. presents some additional data, most notably on the involvement of RNAses in the gal operon regulation, and remove other pieces of data that were questioned by the reviewers. The manuscript is easier to follow and provides some interesting insights into gal control. It is customary, however, to ask if the authors have addressed all the reviewers’ concerns. This is not the case with mine.
I am still not convinced that the data on Rho have any novelty. It is encouraging that Rho terminates transcription when RNAP is paused at a consensus site, since this is consistent with what we know. But there does not seem to be any direct interplay between Spot 42 and Rho. The authors mention a possibility that Spot 42 RNA binding to region 1 could have an effect on termination (incl. in Figure 7) but nothing in their data suggests this.

We have removed this sentence from the text and revised Figure 7 accordingly (l. 345-46)

In the first round or review, I wrote: “the authors should inhibit translation of galK by other means to distinguish different aspects of its contribution to gal control”. This would have been a very simple way to test if Spot 42 does anything in addition to inhibiting translation initiation. Since the plasmids are used in the main “extract” assay, a substitution of the galK start codon would take very little effort. In the absence of this, or any other similar evidence, the null hypothesis is that spot42 inhibits translation, just as expected, and this promotes Rho-dependent termination.

This idea of using a *galK* mutant that hampers translation initiation of *galK* would open up a brand new work regarding a new role of sRNA other than translation initiation. Thus, I thank you for suggesting the idea.

Another request that was made by me and other reviewers but was ignored is a need to report error analysis that would indicate that the results are reproducible. I understand that not every reviewer’s comment has to be addressed, as long as a valid reason is offered. I do not, however, think that the lack of evidence in support of reproducibility is acceptable. The figure legends have to state the number of replicates used. If any fold effect is stated (2-fold, 50% etc), it must be accompanied by error analysis. Otherwise, the numbers should be substituted by some qualitative terms.

These suggestions have been incorporated in the text as well as in the figures. All the experiments performed in the study are representative of three individual experiments. As suggested, the caption of Figures with the blots now state the number of replicates used (l.742-43, 758-59, 765-66, 781-82 and 790-91). Error analysis was implemented in case of fold effect.

With respect to 2 different mechanisms of control, what is really happening here is that the inhibition of translation by spot 42 leads to generation of two shorter RNA species, one via premature termination of the nascent RNA (possibly every transcript, if the pause site in question is strong), the other – by ribonuclease processing of RNAs that have been completely made. In both cases, spot 42 job is to make sure that the proximal RNA, and thus proteins, are present in excess. But from the physiological point of view, these two RNAs should be identical, as their ends are closely spaced. So the outcome is actually the same, just the means differ.

I totally agree with you on these remarks. Thank you. Regarding your ideas, I think that Spot 42 binding inhibits translation initiation of an ordinary ribosome that results in *galET-long*. But Spot 42 binding inhibits translation initiation of the leading ribosome that would result in *galET-short*. I am planning experiments including using the *galK* mutant of translation initiation to show all these ideas.

I suggest making the following changes to Figure 7:
Panel A: remove region I base pairing from the picture and draw ribosome at a real scale = it protects ~23 nt and depicting this correctly would help to show why base pairing in region II does its job
Panel B: in late 2020, papers showing that Rho travels with RNAP and terminates transcription when the enzyme pauses and RNA is captured were published. They should be cited, and the cartoon should reflect these new data.

These suggestions have also been incorporated in the Figure 7A and B. The papers referring to the above mentioned have been cited in the manuscript (l. 342 and 344).

Reviewer #4: The authors have done a good job addressing most the reviewer’s comments and have added new results describing how another well-characterized RNase (RNase III) serves a function generating galET-short. This new finding adds to the authors’ story of how different binding events and parts of Spot 42 RNA potentiate different galET processing events. The authors are suitably careful to leave open the possibility that RNase III processing may be the result of dsRNA that forms intramolecularly vs. between gal and Spot 42—this possibility is acknowledged in the model figure. However, two major, related points raised in the initial review remain unaddressed after revision. These unaddressed points concern reproducibility of the results in the form of enumerating the number of replicates performed or their statistical analysis. Addressing these points will greatly strengthen the conclusions presented in this manuscript.

Major points

1. The authors need to include information regarding the number of replicates performed for representative blot presented in the manuscript. This need could be met simply by stating in the figure captions: “The data presented in these blots are representative of X independent experiments.”

All the experiments performed in the study are representative of three individual experiments. As suggested, the caption of Figures with the blots now state: “The data presented in these blots are representative of three independent experiments (l.742-43, 758-59, 765-66, 781-82 and 790-91).”

2. Fig 6B. Error bars are depicted, but it remains unclear from the text and figure legend what these error bars represent. Presumably, these are the same errors that are propagated to determine the errors in the calculated doubling times presented in Table 1. The error analysis must be fully explained. Are they standard deviations of replicate data? Standard errors of means? The authors should also specify “n=x”, where x is the number of replicates.

We apologize for the confusion, the error bars represents the mean fold-change ± standard deviation of three replicates from three independent experiments (n=3). The error bars representation has been added to the Figure 6B caption (l.799-800).

Minor point

1. In the Figure 7 caption, it would be better to state directly that “RNase (?)” represents currently unidentified RNases(s) that may be involved in the further processing events of the galET transcripts.

As suggested, the Figure 7 caption has been updated (l.819-20).
